# Changes in stroke and TIA admissions during the COVID-19 pandemic: A meta-analysis

**DOI:** 10.1177/23969873231204127

**Published:** 2023-09-29

**Authors:** Karin Gunnarsson, Avin Tofiq, Alen Mathew, Yang Cao, Mia von Euler, Jakob O Ström

**Affiliations:** 1 Department of Neurology and rehabilitation, Faculty of Medicine and Health, School of Medical Sciences, Örebro University, Örebro, Sweden; 2 Clinical Epidemiology and Biostatistics, Faculty of Medicine and Health, School of Medical Sciences, Örebro University, Örebro, Sweden; 3 Unit of Integrative Epidemiology, Institute of Environmental Medicine, Karolinska Institutet, Stockholm, Sweden

**Keywords:** Acute ischemic stroke, cerebral infarction, Corona virus, TIA, intracerebral hemorrhage, NIHSS, SARS-CoV-2

## Abstract

**Purpose:**

To perform a meta-analysis on how the admissions of stroke and transient ischemic attack (TIA) changed during the Corona Virus infection-19 (COVID-19) pandemic and evaluate if the effect was depending on stroke severity.

**Methods:**

Observational cohort studies comparing the number of stroke and/or TIA admissions during a period of the pandemic compared to a period before the pandemic were identified in PubMed and Embase. After excluding studies with overlapping populations and studies without satisfactory case ascertainment, data was extracted and meta-analyzed.

**Findings:**

A total of 59 studies were included. During the pandemic, there was a decrease in admissions of ischemic stroke (admission rate ratio (ARR) = 0.77, 95% confidence interval (CI): 0.72, 0.82), intracerebral hemorrhage (ARR = 0.79, 95% CI: 0.70, 0.90) and TIA (ARR = 0.66, 95% CI: 0.58, 0.75). Albeit admission rates of both mild (ARR = 0.61, 95% CI: 0.49, 0.77) and severe (ARR = 0.82, 95% CI = 0.71, 0.95) strokes decreased, milder strokes decreased more (proportion ratio (PR) = 0.76, 95% CI: 0.65, 0.89).

**Discussion:**

Potential causes for the admission reduction could be strict prioritizations within the health care, patients’ fear of acquiring COVID-19, or decreased access to health care due to lockdowns.

**Conclusion:**

During the COVID-19 pandemic, there was a reduction in admissions of stroke and TIA, possibly caused by reluctance to seek medical care.

## Graphical abstract

**Graphical abstract:**
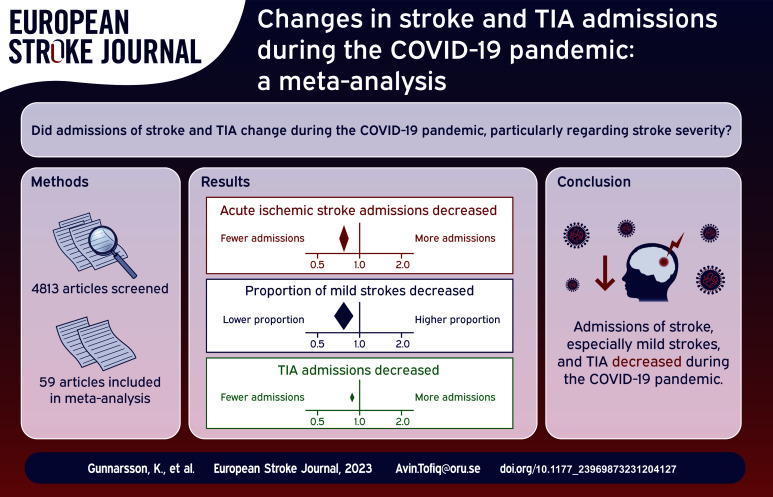

## Introduction

The Corona Virus Disease-19 (COVID-19) pandemic put a great burden on healthcare systems worldwide. This affected the care of many other diseases, such as the acute care of acute coronary syndrome and stroke.^1^ There have been reports on a lower incidence of stroke presentations during the pandemic compared to previous years, especially when studying minor strokes and transient ischemic attacks (TIA).^2^ There also seemed to be a reduction in the number of stroke mimics.^2^ This could reflect a reluctance to seek medical care during the pandemic, either due to fear of contracting COVID-19 or to avoid straining an already burdened healthcare system.^3^ If so, one could hypothesize that the incidence of milder strokes would be more affected than the incidence of severe strokes. Similar trends were observed during previous viral outbreaks, such as Middle East Respiratory Syndrome^4^ and the Ebola epidemic.^5^

Earlier systematic reviews and meta-analyses have analyzed changes in stroke admissions during the pandemic,^2,6–8^ but there are many pitfalls in the epidemiologic studies that these analyses are based on, including pandemic-induced changes in catchment area, overlapping study populations and lack of stringency in confirming the stroke diagnoses. It has been shown for example that the admission rate was lower for stroke mimics, but stable for acute ischemic strokes (AIS), intra-cerebral hemorrhages (ICH) and TIAs.^9^ Therefore, we set out to meta-analyze the published material on the effect of the pandemic on stroke admissions, applying stringent selection criteria to minimize the risk of bias. The aim was to answer the following questions:

- How did the pandemic affect the admissions of stroke and TIA?
- Did the pandemic affect stroke admissions uniformly, regardless of severity?

## Method

This systematic review was conducted in accordance with the Preferred Reporting Items for Systematic Reviews and Meta-analysis guidelines (PRISMA),^10^ and the study protocol is available on the International Prospective Register of Ongoing Systematic Reviews (PROSPERO) (CRD42021248216).

### Search strategy and study inclusion

A broad search matrix was drafted with the help of a medical librarian liaison (Supplements I) in order to retrieve all relevant articles. The databases PubMed and Embase were searched using combinations of the following terms: “COVID 19,” “2019 nCoV,” “new coronavirus”, “SARS CoV-2,” “Stroke,” “Cerebral infarction,” “TIA,” “Transient ischemic attack,” “Intracerebral hemorrhage,” “Intracerebral hematoma,” “ICH,” and “Cerebral ischemia.” Article selection and data extraction were performed by three researchers (KG, AM, and AT) independent of each other. In cases of disagreements, a senior researcher was consulted (MvE or JOS).

Observational cohort studies in English published between December 1st, 2019 and December 7th, 2022 were included. Articles of interest were those comparing the number of stroke and/or TIA admissions during a period of the pandemic compared to a period before or very early on in the pandemic. Only articles describing stringent criteria for stroke or TIA diagnoses were included. We therefore included studies where admission diagnosis was confirmed after assessment by neurologist, the diagnosis was confirmed during hospital stay, there was a discharge diagnose, or there was a diagnosis from a stroke register. Articles had to have absolute numbers of patients for calculating the number of AIS, ICH, or TIAs.

Letters to Editor, editorials, reviews, comments, and articles without an exhaustive inclusion of all stroke cases were excluded. Subsequently, articles including only stroke alerts or only patients undergoing revascularization were excluded. To avoid including multiple studies on the same population and to mitigate the risk that the pandemic affected the catchment area, articles without a defined population were excluded. Articles where the authors stated that the catchment area was markedly changed by the pandemic were also excluded. In those cases that a “duplicate” (in regard of study population) was identified, the study with the largest cohort was included. If the cohort was not clearly defined (by, e.g. not describing the area for inclusion) in the article, the corresponding author was contacted for clarification.

### Data extraction

Extracted data included: Name of first author; year of publication; geographical location of the study, including if the study was single or multi center or national; study design; time period for the COVID-19 period and the control period; number of total stroke, AIS, ICH, and TIA; and National Institutes of Health Stroke Scale (NIHSS)-score for total stroke and AIS, as well as number of mild strokes (NIHSS < 5) and severe strokes (NIHSS ⩾ 5).

### Quality control and bias assessment

Quality control and bias assessment of the studies were made by two of the reviewers (KG and AT) using the Newcastle-Ottawa scale^11^ (Supplements II). The scale allocates points in each of the categories: participant selection, comparability, and outcome. Based on this the studies are defined as being of “Good quality,” “Fair quality,” or “Poor quality.” One study was deemed to be of “fair quality,” while the rest were of “good quality.” The included articles altogether rendered enough points to indicate an overall low risk of bias.

### Statistical analyses

Studies with necessary numeric information that can be used to calculate admission rate, proportion, or mean score with their corresponding 95% confidence intervals (CIs) were included in the meta-analysis. Admission rate was calculated as the number of patients divided by the days of the study period. The effects of COVID-19 on patient admission were calculated as admission rate ratio (ARR), proportion ratio (PR), or standardized mean difference (SMD) of the scores between the COVID-19 period and the control period. The degree of heterogeneity between the studies was assessed using *I*^2^ statistic, in which *I*^2^ > 30% was considered to indicate moderate heterogeneity and *I*^2^ > 50% was considered to indicate substantial heterogeneity. Besides, a *p*-value < 0.05 from the non-central chi-squared test for heterogeneity was considered statistically significant. Random-effects models were used to pool the effects from individual studies when heterogeneity was presented. The publication bias was evaluated using the Egger’s test and presented using funnel plots (Supplements III). For the pooled results, a 95% CI that did not include 1 (for ARR and PR) or 0 (for SMD) was considered statistically significant. All analyses were performed in Stata 17.0 (StataCorp, College Station, Texas, USA).

## Findings

### Article inclusion

**Figure 1.**
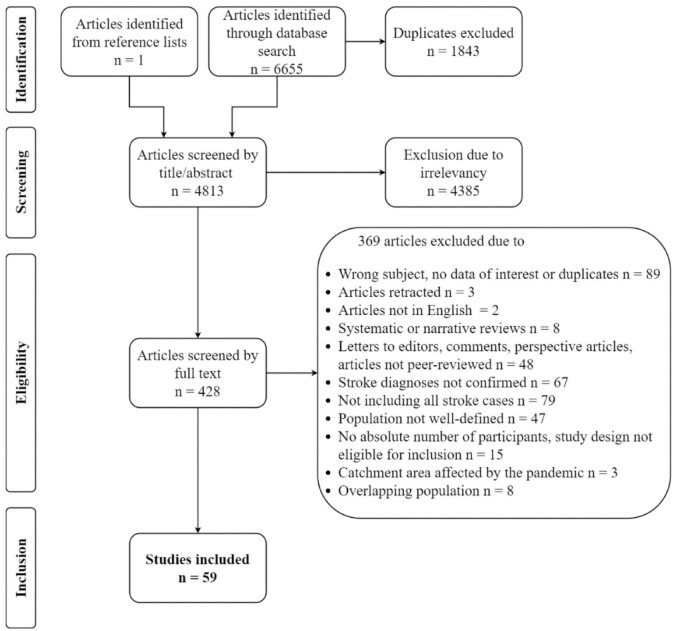
Flowchart over screening and inclusion of studies.

The systematic literature search identified 6655 articles. After screening and exclusion, 59 articles remained and were included in the study (Figure 1).

### Stroke admissions decreased during the pandemic

**Figure 2.**
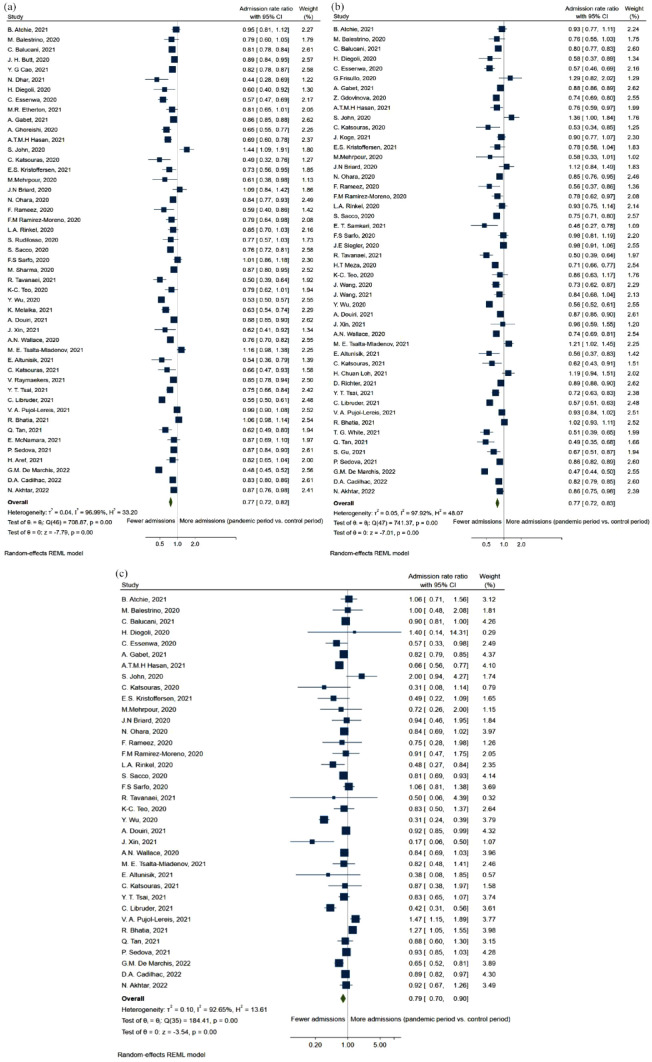
Forest plot of admissions rate ratio between pandemic and pre-pandemic time-periods for (a) stroke, (b) acute ischemic stroke, and (c) intra-cerebral hemorrhage.

A total of 47 studies presented numbers on the total stroke admissions before and during the pandemic, with *n* = 141,818 in the pre-pandemic group and *n* = 59,979 in the pandemic group. Compared to the pre-pandemic period, the rate of stroke admissions decreased during the pandemic (ARR = 0.77, 95% CI: 0.72, 0.82; Figure 2). Similar results were observed for AIS (ARR = 0.77, 95% CI: 0.72, 0.83; Figure 2), with 47 studies presenting results, the pre-pandemic group consisting of *n* = 172,532 and the pandemic group of *n* = 99,870. Only 36 studies reported numbers on ICH, with *n* = 24,454 in the pre-pandemic group and *n* = 9214 in the pandemic group. Here as well, a decrease in admissions rate was observed (ARR = 0.79, 95% CI: 0.70, 0.90; Figure 2).

### Increased average stroke severity due to decreased proportion of mild stroke

**Figure 3.**
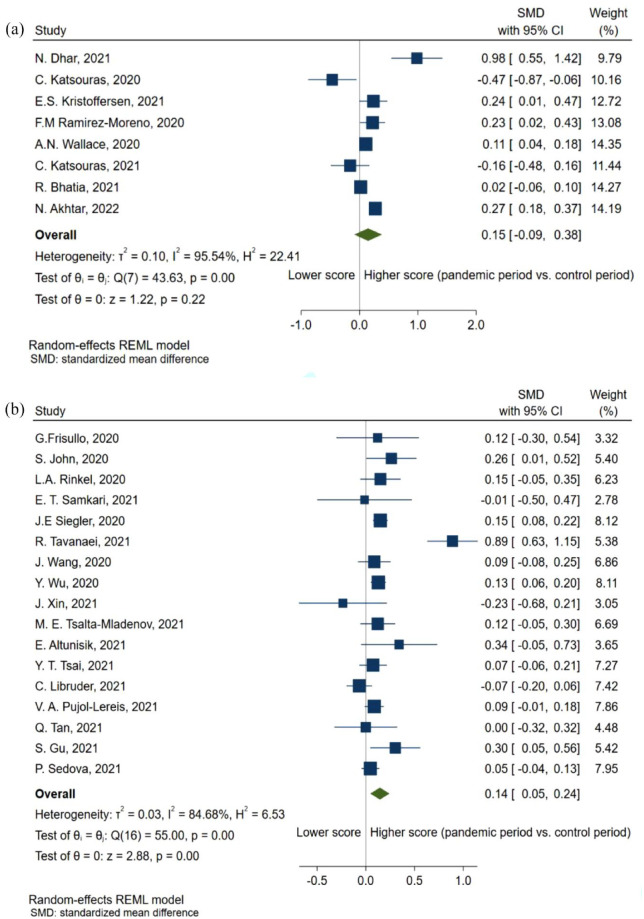
Forest plot of National Institutes of Health Stroke Scale scores standardized mean difference between pandemic and pre-pandemic time-periods in patients with (a) stroke and (b) acute ischemic stroke.

**Figure 4.**
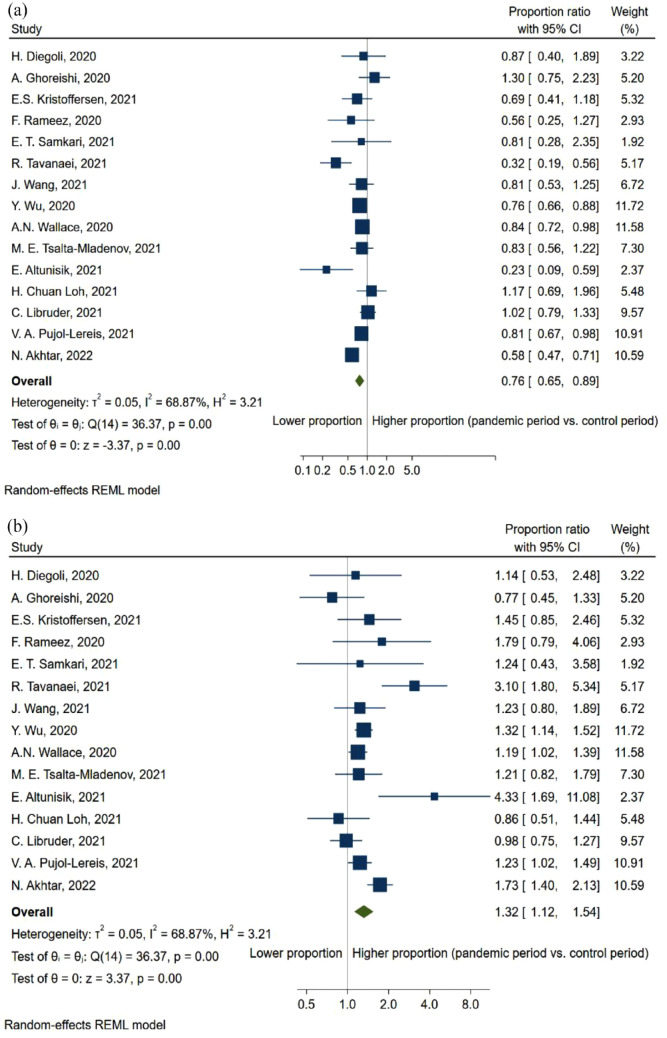
Forest plot of proportion of patients with (a) mild stroke and (b) severe stroke, between pandemic and pre-pandemic time-periods.

**Figure 5.**
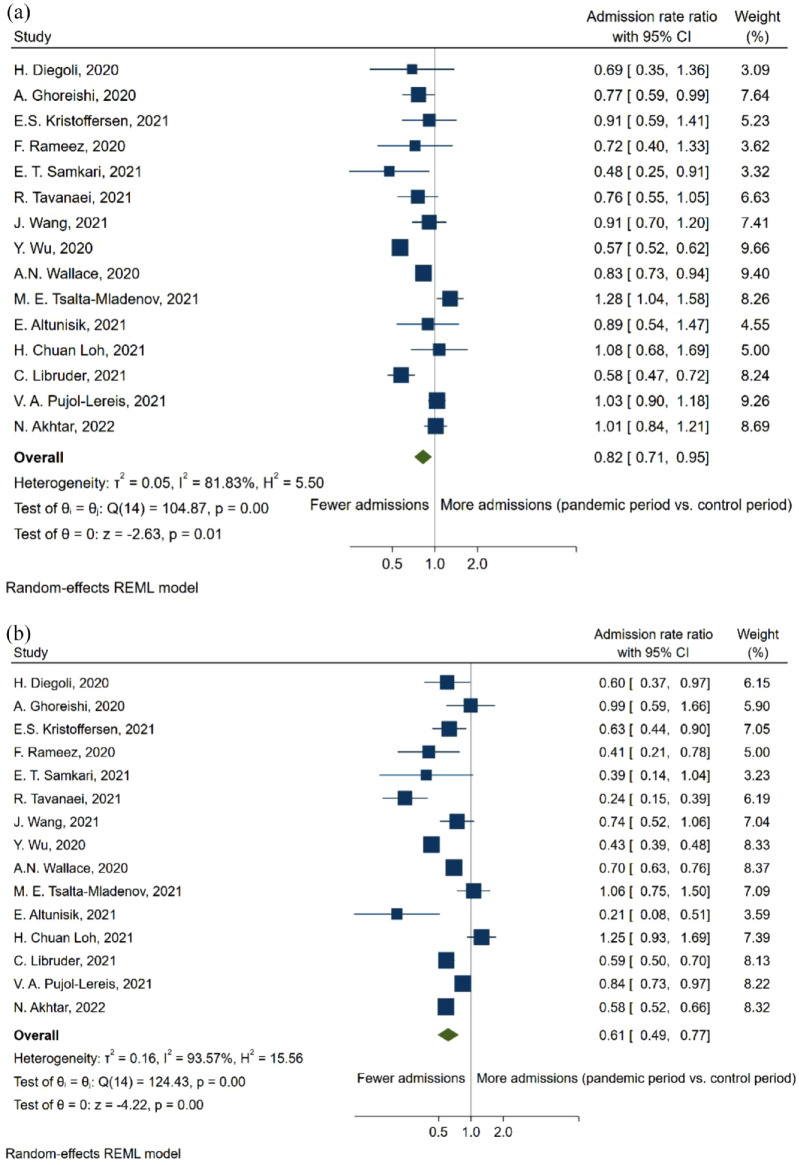
Forest plot of admissions rate ratio between pandemic and pre-pandemic time-periods for (a) severe stroke and (b) mild stroke.

When comparing the NIHSS-score of patients with any type of stroke before and during the pandemic, there was a non-significant tendency toward a higher score (SMD = 0.15, 95% CI: −0.09, 0.38; Figure 3). Furthermore, this trend became significant when only including patients with AIS (SMD = 0.14, 95% CI: 0.05, 0.24; Figure 3). Concurrently, the proportion of reported mild stroke admittance decreased during the pandemic (PR = 0.76, 95% CI: 0.65, 0.89; Figure 4), mirroring an increased proportion of severe strokes (PR = 1.32, 95% CI: 1.12, 1.54; Figure 4) during the pandemic. However, decreases in admission rate were observed in both severe strokes (ARR = 0.82, 95% CI: 0.71, 0.95; Figure 5) and mild strokes (ARR = 0.61, 95% CI: 0.49, 0.77; Figure 5), but with a greater decrease in mild strokes.

### TIA admissions decreased during the pandemic

**Figure 6.**
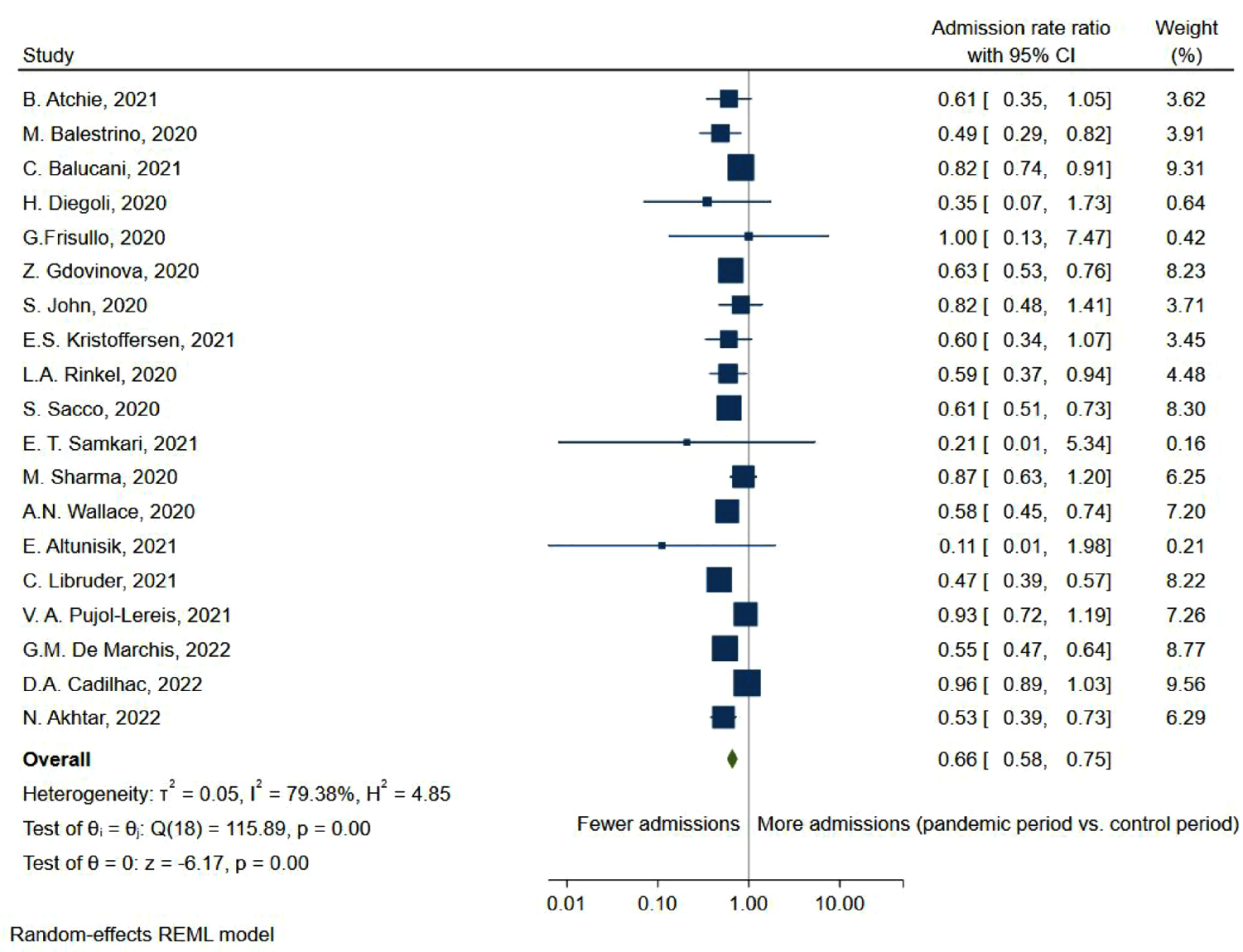
Forest plot of transient ischemic attack admissions rate ratio between pandemic and pre-pandemic time-periods.

Similarly to stroke, the admissions rate of TIA decreased during the pandemic (ARR = 0.66, 95% CI: 0.58, 0.75; Figure 6).

## Discussion

Overall, this meta-analysis shows a 23% reduction in the admissions rate of stroke during the pandemic (Figure 2), while the proportion of mild stroke decreased with 24% (Figure 4), in turn leading to a 0.14-point increase in the standardized mean NIHSS-score (Figure 3). At the same time, the admissions rate of TIA decreased by 34% (Figure 6).

These findings are in line with previous publications,^1,2,6,7^ which have shown reductions in stroke admissions by 30%^7^ and 26%.^2^ Our results point to a slightly more subtle reduction, which could be due to a more robust design with stricter inclusion criteria.

As is always the case with epidemiological studies, strictly proving causality is not possible, and can therefore neither be claimed in the original articles nor in our meta-analysis. With that being said, the clear reduction in stroke admissions during the pandemic does suggest that the pandemic itself indeed was a driver of this decline. Many potential causes for the change in admissions and proportions between mild and severe stroke have been discussed. The strain on the health care system during the pandemic leading to very strict prioritization is one possible cause. Another such reason is that the fear of acquiring a COVID-19-infection might have deterred people with mild symptoms from seeking medical care. This might explain not only the drop in admissions, but also the change in proportions of mild and severe stroke.^3^ Not seeking immediate care for a mild stroke or a TIA is dangerous, as the risk of having another stroke is high,^12^ and secondary prevention can mitigate that risk by up to 80%.^13^

Previous studies have shown that the systemic inflammation caused by COVID-19 may increase the risk of having a stroke^14^ and, as noticed early in the pandemic, an increase in large vessel occlusions.^6^ This meta-analysis cannot be used to draw any conclusions regarding COVID-19 as a risk factor for stroke, but it can at least be concluded that any increased risk of stroke due to COVID-19 in the population was heavily counterbalanced by some other factor, probably reluctance of seeking medical help.

Another reason for the change in admissions that has been proposed is that lockdowns and changes in patient flow with some hospitals being turned into dedicated COVID-19-hospitals might have decreased the publics’ access to healthcare and thereby prevented patients from seeking care.^7^ However, this factor was minimized in the current meta-analysis by excluding studies that have stated that the patient flow and catchment area was affected by the pandemic.

The current meta-analysis has important limitations. One is that the included studies differed in what time periods, pre-pandemic and intra-pandemic, were compared, which slightly hampers comparability between studies. Of the 59 included studies, 15 had control periods that reached into the beginning of 2020, and thus bordering the actual start of the pandemic (Supplements IV). However, since the pandemic affected different countries at different time periods, strictly defined common time periods for all studies would probably have led to an underestimation of the investigated effects. Another limitation is that because of our strict inclusion criteria, including stroke diagnosis ascertainment, several studies could not be included. On the other hand, the very high number of published studies still rendered a substantial number of cases to be meta-analyzed. Further, due to our meta-analytical approach, geographical and socioeconomic disparities in how the pandemic affected stroke admissions might have been lost.^15^ It also means that we are restricted to only studying if there has been a change in stroke and TIA admissions, and not why such a change might have occurred. Further studies are needed in order to answer such questions.

## Conclusion

The COVID-19 pandemic led to a reduction in admissions of stroke and TIA. Although the decreased incidence was seen in both mild and severe strokes, there was a change in the proportion of mild and severe stroke, with an increase in the proportion of severe stroke. Further studies are needed to see how this change might affect the incidence of stroke, the admissions and the proportions between mild and severe stroke after the pandemic.

## Supplemental Material

sj-docx-1-eso-10.1177_23969873231204127 – Supplemental material for Changes in stroke and TIA admissions during the COVID-19 pandemic: A meta-analysis Supplemental material, sj-docx-1-eso-10.1177_23969873231204127 for Changes in stroke and TIA admissions during the COVID-19 pandemic: A meta-analysis by Karin Gunnarsson, Avin Tofiq, Alen Mathew, Yang Cao, Mia von Euler and Jakob O Ström in European Stroke Journal

sj-docx-2-eso-10.1177_23969873231204127 – Supplemental material for Changes in stroke and TIA admissions during the COVID-19 pandemic: A meta-analysis Supplemental material, sj-docx-2-eso-10.1177_23969873231204127 for Changes in stroke and TIA admissions during the COVID-19 pandemic: A meta-analysis by Karin Gunnarsson, Avin Tofiq, Alen Mathew, Yang Cao, Mia von Euler and Jakob O Ström in European Stroke Journal

sj-docx-3-eso-10.1177_23969873231204127 – Supplemental material for Changes in stroke and TIA admissions during the COVID-19 pandemic: A meta-analysis Supplemental material, sj-docx-3-eso-10.1177_23969873231204127 for Changes in stroke and TIA admissions during the COVID-19 pandemic: A meta-analysis by Karin Gunnarsson, Avin Tofiq, Alen Mathew, Yang Cao, Mia von Euler and Jakob O Ström in European Stroke Journal

sj-docx-4-eso-10.1177_23969873231204127 – Supplemental material for Changes in stroke and TIA admissions during the COVID-19 pandemic: A meta-analysis Supplemental material, sj-docx-4-eso-10.1177_23969873231204127 for Changes in stroke and TIA admissions during the COVID-19 pandemic: A meta-analysis by Karin Gunnarsson, Avin Tofiq, Alen Mathew, Yang Cao, Mia von Euler and Jakob O Ström in European Stroke Journal
