# Supplementary material for: Changes in stroke and TIA admissions during the COVID-19 pandemic: A meta-analysis
Source: Eur Stroke J. 2023 Sep 29;9(1):78–87. doi: 10.1177/23969873231204127 (PMC10916820; doi:10.1177/23969873231204127)
Supplement: sj-docx-1-eso-10.1177_23969873231204127 – Supplemental material for Changes in stroke and TIA admissions during the COVID-19 pandemic: A meta-analysis [file sj-docx-1-eso-10.1177_23969873231204127.docx]

Pubmed

| Stroke | | |
| --- | --- | --- |
|  | #1 | "Stroke"[Mesh] OR "Ischemic Attack, Transient"[Mesh] |
|  | #2 | stroke*[Title/Abstract] OR "cerebrovascular accident*"[Title/Abstract] OR "cva"[Title/Abstract] OR cvas[Title/Abstract] OR apoplex*[Title/Abstract] OR "brain vascular accident*"[Title/Abstract] OR "brain infarct*"[Title/Abstract] OR "cerebral infarct*"[Title/Abstract] OR tia[Title/Abstract] OR tias[Title/Abstract] OR "Transient Ischemic Attack"[Title/Abstract] OR "Transient Ischemic Attacks"[Title/Abstract] OR "Transient Cerebral Ischemia"[Title/Abstract] OR "Transient Cerebral Ischemias"[Title/Abstract] OR "Intracerebral hemorrhage"[Title/Abstract] OR "Intracerebral haemorrhage"[Title/Abstract] OR "intracerebral hematoma"[Title/Abstract] OR "intracerebral haematoma"[Title/Abstract] OR "ICH"[Title/Abstract] OR "cerebral ischemia*"[Title/Abstract] OR "cerebral ischaemia*"[Title/Abstract] |
|  | #3 | #1 OR #2 |
| Covid 19 | | |
|  | #4 | "COVID-19"[Mesh] OR "SARS-CoV-2"[Mesh] OR "SARS-CoV-2 variants" [Supplementary Concept] OR "post-acute COVID-19 syndrome" [Supplementary Concept] OR "Coronavirus"[Mesh] OR "Coronavirus Infections"[Mesh] |
|  | #5 | coronavir*[Title/Abstract] OR "corona virus*"[Title/Abstract] OR "corona viri*"[Title/Abstract] OR nCov*[Title/Abstract] OR covid*[Title/Abstract] OR "SARS COV"[Title/Abstract] OR sarscov*[Title/Abstract] OR "Sars-coronavirus*"[Title/Abstract] OR "Severe Acute Respiratory Syndrome Coronavirus*"[Title/Abstract] OR "2019 ncov"[Title/Abstract] OR ncov19[Title/Abstract] OR ncov-19[Title/Abstract] OR "2019-novel CoV"[Title/Abstract] OR 2019-ncov[Title/Abstract] OR nCov-2019[Title/Abstract] OR "CoV 2"[Title/Abstract] OR CoV-2[Title/Abstract] OR "SARS-like coronavirus*"[Title/Abstract] OR betacoronavirus[Title/Abstract] OR betacoronaviruses[Title/Abstract] OR OC43[Title/Abstract] OR NL63[Title/Abstract] OR 229E[Title/Abstract] OR HKU1[Title/Abstract] OR HCoV*[Title/Abstract] OR cov[Title/Abstract] |
|  | #6 | #4 OR #5 |
| Stroke and Covid 19 combined | | |
|  | #7 | #3 AND #6 |

Embase

| Search terms | | |
| --- | --- | --- |
| Stroke | | |
|  | 1 | 'cerebrovascular accident'/exp OR 'stroke patient'/exp |
|  | 2 | 'transient ischemic attack'/exp |
|  | 3 | stroke*:ab,ti,kw OR 'cerebrovascular accident*':ab,ti,kw OR cva:ab,ti,kw OR cvas:ab,ti,kw OR apoplex*:ab,ti,kw OR 'brain vascular accident*':ab,ti,kw OR 'brain infarct*':ab,ti,kw OR 'cerebral infarct*':ab,ti,kw OR tia:ab,kw,ti OR tias:ab,kw,ti OR 'transient ischemic attack':ab,kw,ti OR 'transient ischemic attacks':ab,kw,ti OR 'transient cerebral ischemia':ab,kw,ti OR 'transient cerebral ischemias':ab,kw,ti OR 'intracerebral hemorrhage':ab,kw,ti OR 'intracerebral haemorrhage':ab,kw,ti OR 'intracerebral hematoma':ab,kw,ti OR 'intracerebral haematoma':ab,kw,ti OR 'ich':ab,kw,ti OR 'cerebral ischemia*':ab,kw,ti OR 'cerebral ischaemia*':ab,kw,ti |
|  | 4 | 1 OR 2 OR 3 |
| Covid 19 | | |
|  | 5 | 'coronavirus infection'/exp |
|  | 6 | 'coronavirinae'/exp |
|  | 7 | 'severe acute respiratory syndrome coronavirus 2'/exp |
|  | 8 | coronavir*:ab,kw,ti OR 'corona vir*':ab,kw,ti OR ncov*:ab,kw,ti OR covid*:ab,kw,ti OR 'sars cov*':ab,kw,ti OR sarscov*:ab,kw,ti OR 'sars-coronavirus*':ab,kw,ti OR 'severe acute respiratory syndrome coronavirus*':ab,kw,ti OR ncov:ab,kw,ti OR ncov19:ab,kw,ti OR 'ncov 19':ab,kw,ti OR '2019-novel cov':ab,kw,ti OR '2019 ncov':ab,kw,ti OR 'ncov 2019':ab,kw,ti OR 'cov 2':ab,kw,ti OR 'sars-like coronavirus*':ab,kw,ti OR betacoronavirus:ab,kw,ti OR betacoronaviruses:ab,kw,ti OR oc43:ab,kw,ti OR nl63:ab,kw,ti OR 229e:ab,kw,ti OR hku1:ab,kw,ti OR hcov*:ab,kw,ti OR cov:ab,kw,ti |
|  | 9 | 5 OR 6 OR 7 OR 8 |
| Blocks combined | | |
|  | 10 | 4 AND 9 |
