# Supplementary material for: Changes in stroke and TIA admissions during the COVID-19 pandemic: A meta-analysis
Source: Eur Stroke J. 2023 Sep 29;9(1):78–87. doi: 10.1177/23969873231204127 (PMC10916820; doi:10.1177/23969873231204127)
Supplement: sj-docx-2-eso-10.1177_23969873231204127 – Supplemental material for Changes in stroke and TIA admissions during the COVID-19 pandemic: A meta-analysis [file sj-docx-2-eso-10.1177_23969873231204127.docx]

| **Newcastle-Ottawa Quality Assessment Scale** | | | | | | | | | | | | | | |
| --- | --- | --- | --- | --- | --- | --- | --- | --- | --- | --- | --- | --- | --- | --- |
| **Article information** | | | | | **Selection** | | | | **Comparability** | | **Outcome** | | | **Overall score** |
| **PMID** | **First author** | **Published** | **Study type** | **Region** | **1** | **2** | **3** | **4** | **1a** | **1b** | **1** | **2** | **3** |  |
| 33758066 | B. Atchie | 210323 | Retrospective cohort study | USA | 1 | 1 | 1 | 1 | 1 | 1 | 0 | 1 | 0 | 7/9 |
| 33063236 | M. Balestrino | 201015 | Retrospective cohort study | Italy, Genoa | 1 | 1 | 1 | 1 | 1 | 1 | 0 | 1 | 0 | 7/9 |
| 33691503 | C. Balucani | 210311 | Retrospective cohort study | US, Maryland | 1 | 1 | 1 | 1 | 1 | 0 | 1 | 1 | 0 | 7/9 |
| 32755320 | J. H. Butt | 200805 | Retrospective cohort study | Denmark | 1 | 1 | 1 | 1 | 1 | 1 | 1 | 1 | 0 | 8/9 |
| 33523320 | Y. G Cao | 210201 | Retrospective cohort study | China, Wenzhou | 1 | 1 | 1 | 1 | 1 | 1 | 1 | 1 | 0 | 8/9 |
| 34295099 | N. Dhar | 210222 | Retrospective cohort study | India, Uttrakand | 1 | 1 | 1 | 1 | 1 | 0 | 1 | 1 | 1 | 8/9 |
| 32530738 | H. Diegoli | 200612 | Retrospective cohort study | Brazil, Joinville | 1 | 1 | 1 | 1 | 1 | 0 | 1 | 1 | 1 | 8/9 |
| 32912527 | C. Essenwa | 200713 | Retrospective cohort study | USA, NY | 1 | 1 | 1 | 1 | 1 | 0 | 1 | 1 | 1 | 8/9 |
| 33588592 | M.R. Etherton | 210216 | Retrospective cohort study | USA, New England | 1 | 1 | 1 | 1 | 1 | 0 | 1 | 1 | 0 | 7/9 |
| 32632635 | G.Frisullo | 200706 | Retrospective cohort study | Italy, Lazio | 1 | 1 | 1 | 1 | 1 | 1 | 1 | 1 | 1 | 9/9 |
| 33738913 | A. Gabet | 210329 | Retrospective cohort study | France, national | 1 | 1 | 1 | 1 | 1 | 1 | 1 | 1 | 1 | 9/9 |
| 33185918 | Z. Gdovinova | 201203 | Retrospective cohort study | Slovakia | 1 | 1 | 1 | 1 | 1 | 1 | 1 | 1 | 1 | 9/9 |
| 33069086 | A. Ghoreishi | 201201 | Retrospective cohort study | Iran | 1 | 1 | 1 | 1 | 1 | 1 | 0 | 1 | 0 | 7/9 |
| 33439890 | A.T.M.H Hasan | 210113 | Retrospective cohort study | Bangladesh | 1 | 1 | 1 | 1 | 1 | 0 | 1 | 1 | 0 | 7/9 |
| 33011516 | S. John | 201111 | Retrospective cohort study | UAE, Abu Dhabi | 1 | 1 | 1 | 1 | 1 | 1 | 0 | 1 | 1 | 8 /9 |
| 33290619 | C. Katsouras | 201227 | Retrospective cohort study | Greece | 1 | 1 | 1 | 1 | 1 | 1 | 0 | 1 | 0 | 7/9 |
| 33536996 | J. Koge | 210118 | Retrospective cohort study | Japan | 1 | 1 | 1 | 1 | 1 | 0 | 0 | 1 | 0 | 6/9 |
| 33428057 | E.S. Kristoffersen | 210111 | Retrospective cohort study | Norway | 1 | 1 | 1 | 1 | 1 | 0 | 1 | 1 | 1 | 8/9 |
| 32510283 | M.Mehrpour | 200626 | Retrospective cohort study | Iran | 1 | 1 | 1 | 1 | 1 | 1 | 1 | 1 | 1 | 9/9 |
| 32698917 | J.N Briard | 200723 | Retrospective cohort study | Canada, Montreal | 1 | 1 | 1 | 1 | 1 | 1 | 1 | 1 | 1 | 9/9 |
| 33039766 | N. Ohara | 200930 | Retrospective cohort study | Japan, Kobe | 1 | 1 | 1 | 1 | 1 | 1 | 0 | 1 | 0 | 7/9 |
| 33166950 | F. Rameez | 201109 | Retrospective cohort study | USA, Michigan | 1 | 1 | 1 | 1 | 1 | 1 | 0 | 1 | 0 | 7/9 |
| 33450843 | F.M Ramirez-Moreno | 210113 | Retrospective cohort study | Spain | 1 | 1 | 1 | 1 | 1 | 1 | 0 | 1 | 0 | 7/9 |
| 32691235 | L.A. Rinkel | 200720 | Retrospective cohort study | Amsterdam, The Netherlands | 1 | 1 | 1 | 1 | 1 | 0 | 1 | 1 | 1 | 8/9 |
| 32438895 | S. Rudilosso | 200522 | Retrospective cohort study | Spain, Barcelona | 1 | 1 | 1 | 1 | 1 | 1 | 1 | 1 | 0 | 8/9 |
| 33059543 | S. Sacco | 201016 | Retrospective cohort study | Italy | 1 | 1 | 1 | 1 | 1 | 1 | 1 | 1 | 1 | 9/9 |
| 33814368 | E. T. Samkari | 210426 | Retrospective cohort study | Saudiarabien, Makkah | 1 | 1 | 1 | 1 | 1 | 0 | 1 | 1 | 1 | 8/9 |
| 32702560 | F.S Sarfo | 200915 | Retrospective cohort study | Ghana, Kumasi | 1 | 1 | 1 | 1 | 1 | 1 | 1 | 1 | 1 | 9/9 |
| 32855352 | M. Sharma | 200827 | Retrospective cohort study |  | 1 | 1 | 1 | 1 | 1 | 1 | 0 | 1 | 0 | 7/9 |
| 33250041 | J.E Siegler | 201130 | Retrospective cohort study | USA, | 1 | 1 | 1 | 1 | 1 | 1 | 1 | 1 | 0 | 8/9 |
| 33394194 | R. Tavanaei | 210104 | Retrospective cohort study | Iran, Teheran | 1 | 1 | 1 | 1 | 1 | 1 | 1 | 1 | 1 | 9/9 |
| 32525468 | H.T Meza | 200626 | Retrospective cohort study | Spain | 1 | 1 | 1 | 1 | 1 | 0 | 1 | 1 | 1 | 8/9 |
| 32432998 | K-C. Teo | 200520 | Retrospective cohort study | Hong Kong | 1 | 1 | 1 | 1 | 1 | 1 | 1 | 1 | 0 | 8/9 |
| 33049464 | J. Wang | 200925 | Retrospective cohort study | US,Virginia,Maryland | 1 | 1 | 1 | 1 | 1 | 0 | 1 | 1 | 0 | 7/9 |
| 33550778 | J. Wang | 210625 | Retrospective cohort study | China, Sichuan | 1 | 1 | 1 | 1 | 1 | 1 | 1 | 1 | 0 | 8/9 |
| 33250851 | Y. Wu | 201105 | Retrospective cohort study | China, Beijing | 1 | 1 | 1 | 1 | 1 | 1 | 1 | 1 | 1 | 9/9 |
| 33672096 | K. Melaika | 210223 | Retrospective cohort study | Lithuainia | 1 | 1 | 1 | 1 | 1 | 0 | 1 | 1 | 1 | 8/9 |
| 33896223 | A. Douiri | 210426 | Retrospective cohort study | UK, England, Wales, NI | 1 | 1 | 1 | 1 | 1 | 1 | 1 | 1 | 1 | 9/9 |
| 33789627 | J. Xin | 2021 | Retrospective cohort study | China, Fujian province | 1 | 1 | 1 | 1 | 1 | 1 | 1 | 1 | 1 | 9/9 |
| 33862541 | A.N. Wallace | 2020 | Retrospective cohort study | US, nätverk av sjukhus | 1 | 1 | 1 | 1 | 1 | 0 | 1 | 1 | 0 | 7/9 |
| Embase | M. E. Tsalta-Mladenov | 210921 | Retrospective cohort study | Bulgaria | 1 | 1 | 1 | 1 | 1 | 1 | 1 | 1 | 1 | 9/9 |
| Embase | E. Altunisik | accepted 210108 | Retrospective cohort study | Turkiet | 1 | 1 | 1 | 1 | 1 | 1 | 0 | 1 | 0 | 7/9 |
| 34285718 | C. Katsouras | 210707 | Retrospective cohort study | Grekland (southern: Athens; northern: Thessaloniki; western: Ioannina) | 1 | 1 | 1 | 1 | 1 | 1 | 0 | 1 | 0 | 7/9 |
| 34069433 | H. Chuan Loh | 210519 | Retrospective cohort study | Malaysia | 1 | 1 | 1 | 1 | 1 | 1 | 1 | 1 | 1 | 9/9 |
| 34148220 | V. Raymaekers | 210619 | Retrospective cohort study | Belgien | 1 | 1 | 1 | 1 | 1 | 1 | 0 | 1 | 0 | 7/9 |
| 34370900 | D. Richter | 210809 | Retrospective cohort study | Tyskland | 1 | 1 | 1 | 1 | 1 | 1 | 1 | 1 | 1 | 9/9 |
| Embase | Y. T. Tsai | 210508 acceptance | Retrospective cohort study | Taiwan, Yunlin | 1 | 1 | 1 | 1 | 1 | 1 | 1 | 1 | 0 | 8/9 |
| 34237727 | C. Libruder | 210708 | Retrospective cohort study | Israel | 1 | 1 | 1 | 1 | 1 | 0 | 1 | 1 | 0 | 7/9 |
| 34284323 | V. A. Pujol-Lereis | 210901 | Retrospective cohort study | Sydamerika | 1 | 1 | 1 | 1 | 1 | 1 | 1 | 1 | 0 | 8/9 |
| 34375915 | R. Bhatia | 210915 | Retrospective cohort study | Indien | 1 | 1 | 1 | 1 | 1 | 1 | 1 | 1 | 0 | 8/9 |
| 34336181 | T. G. White | 210729 | Retrospective cohort study | USA, NY | 1 | 1 | 1 | 1 | 1 | 1 | 1 | 1 | 0 | 8/9 |
| 34566833 | Q. Tan | 210908 | Retrospective cohort study | China, Chongqing | 1 | 1 | 1 | 1 | 1 | 1 | 1 | 1 | 0 | 8/9 |
| 34247153 | S. Gu | 210709 | Retrospective cohort study | China, Jiangsu | 1 | 1 | 1 | 1 | 1 | 0 | 1 | 1 | 1 | 8/9 |
| 34168026 | E. McNamara | 210624 | Retrospective cohort study | Australien | 1 | 1 | 1 | 1 | 1 | 1 | 1 | 1 | 1 | 9/9 |
| 34515067 | P. Sedova | 210811 | Retrospective cohort study | Tjeckien | 1 | 1 | 1 | 1 | 1 | 0 | 1 | 1 | 1 | 8/9 |
| 34260632 | H. Aref | 210714 | Prospective cohort study | Egypt | 1 | 1 | 1 | 1 | 1 | 0 | 1 | 1 | 1 | 8/9 |
| 34894018 | G.M. De Marchis | 211211 | Retrospective cohort study | Switzerland | 1 | 1 | 1 | 1 | 1 | 1 | 1 | 1 | 0 | 8/9 |
| 35135062 | D.A. Cadilhac | 220131 | Retrospective cohort study | Australia | 1 | 1 | 1 | 1 | 1 | 0 | 1 | 1 | 0 | 7/9 |
| 35324905 | N. Akhtar | 220324 | Retrospective cohort study | Qatar | 1 | 1 | 1 | 1 | 1 | 0 | 1 | 1 | 0 | 7/9 |
| **Sum of points** | | | | | 59 | 59 | 59 | 59 | 59 | 39 | 46 | 59 | 27 | 466/531 |
