# Supplementary material for: Changes in stroke and TIA admissions during the COVID-19 pandemic: A meta-analysis
Source: Eur Stroke J. 2023 Sep 29;9(1):78–87. doi: 10.1177/23969873231204127 (PMC10916820; doi:10.1177/23969873231204127)
Supplement: sj-docx-3-eso-10.1177_23969873231204127 – Supplemental material for Changes in stroke and TIA admissions during the COVID-19 pandemic: A meta-analysis [file sj-docx-3-eso-10.1177_23969873231204127.docx]

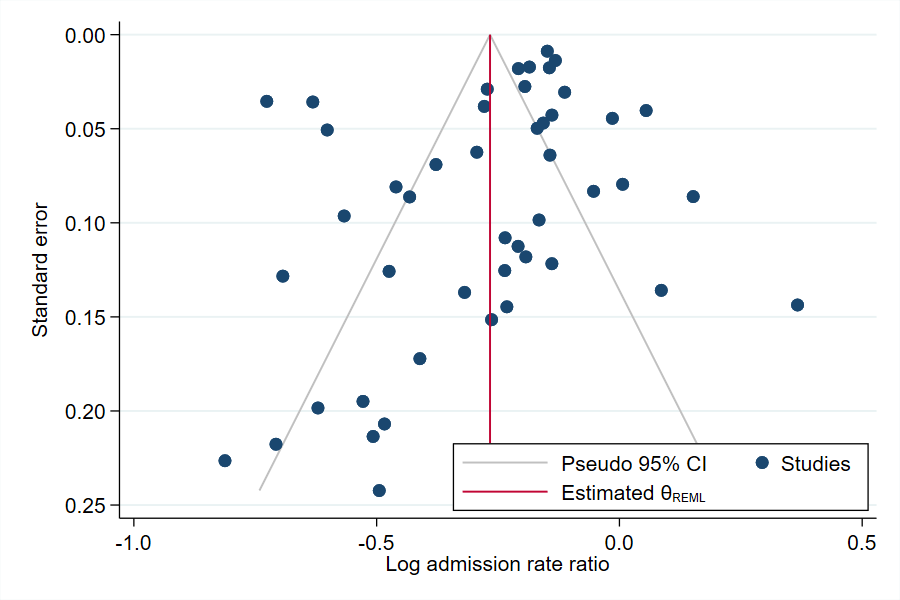


Figure 1. Funnel plot of articles studying stroke admissions between pandemic and pre-pandemic time-periods.


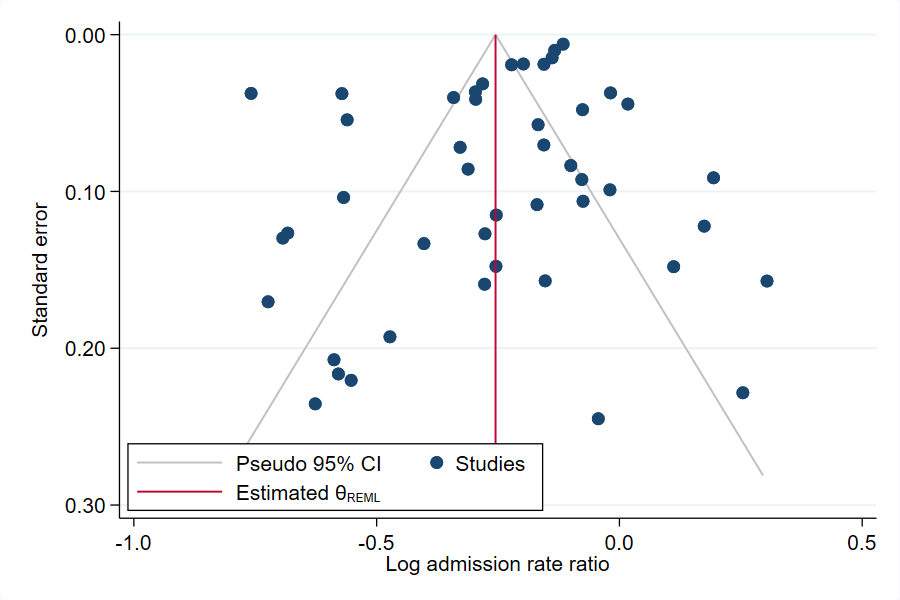


Figure 2. Funnel plot of articles studying acute ischemic stroke admissions between pandemic and pre-pandemic time-periods.


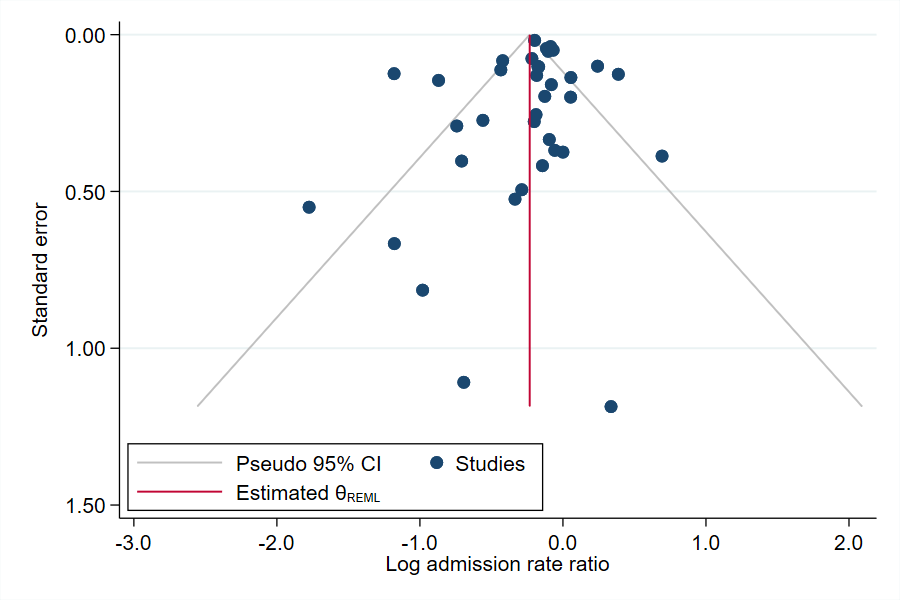


Figure 3. Funnel plot of articles studying intra-cerebral haemorrhage admissions between pandemic and pre-pandemic time-periods.


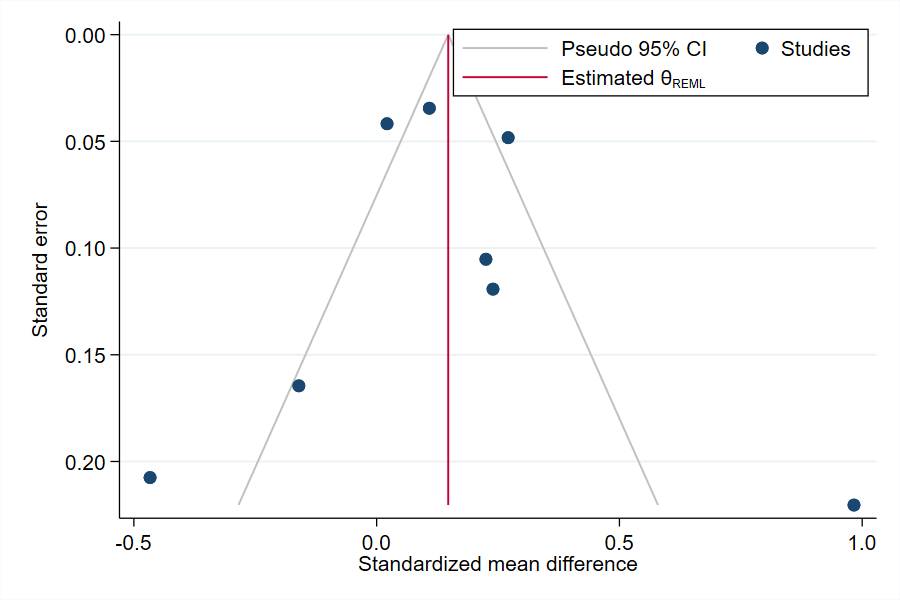


Figure 4. Funnel plot of articles studying National Institutes of Health Stroke Scale scores in patients with stroke between pandemic and pre-pandemic time-periods.


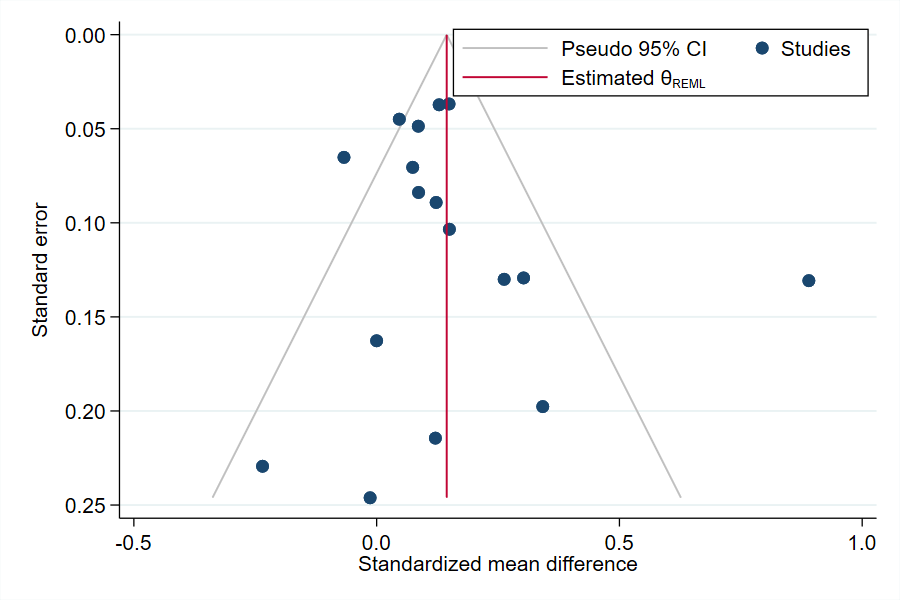


Figure 5. Funnel plot of articles studying National Institutes of Health Stroke Scale scores in patients with acute ischemic stroke between pandemic and pre-pandemic time-periods.


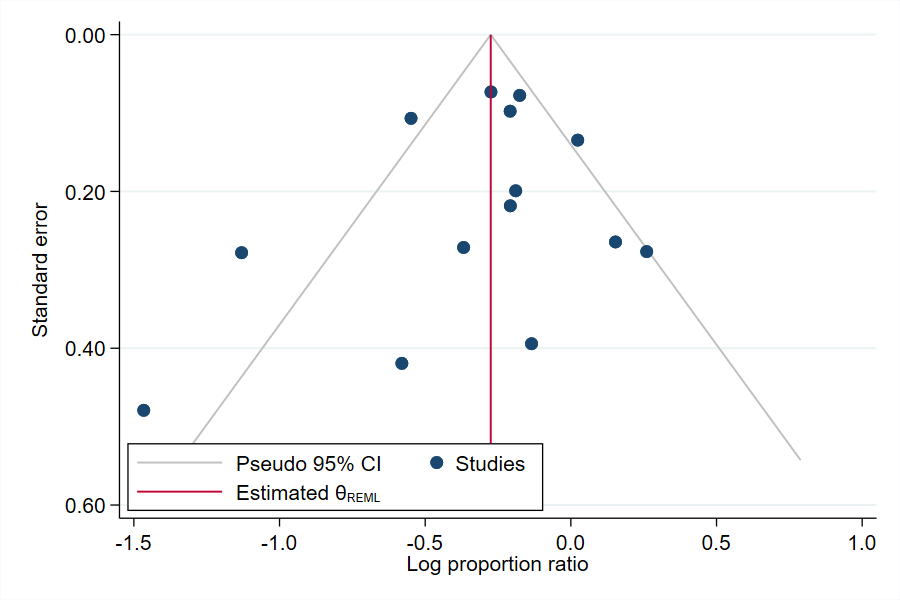


Figure 6. Funnel plot of articles studying proportion of patients with mild stroke between pandemic and pre-pandemic time-periods.


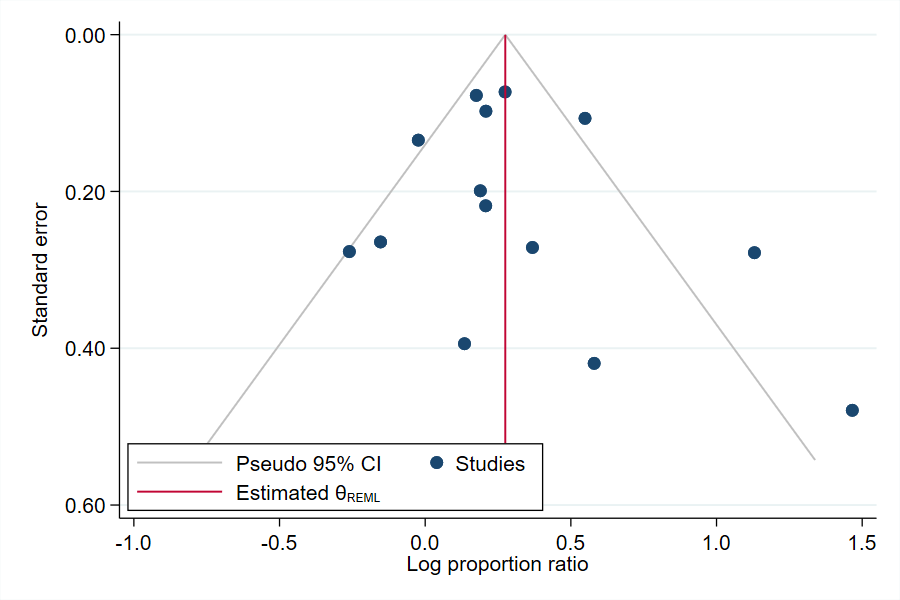


Figure 7. Funnel plot of articles studying proportion of patients with severe stroke between pandemic and pre-pandemic time-periods.


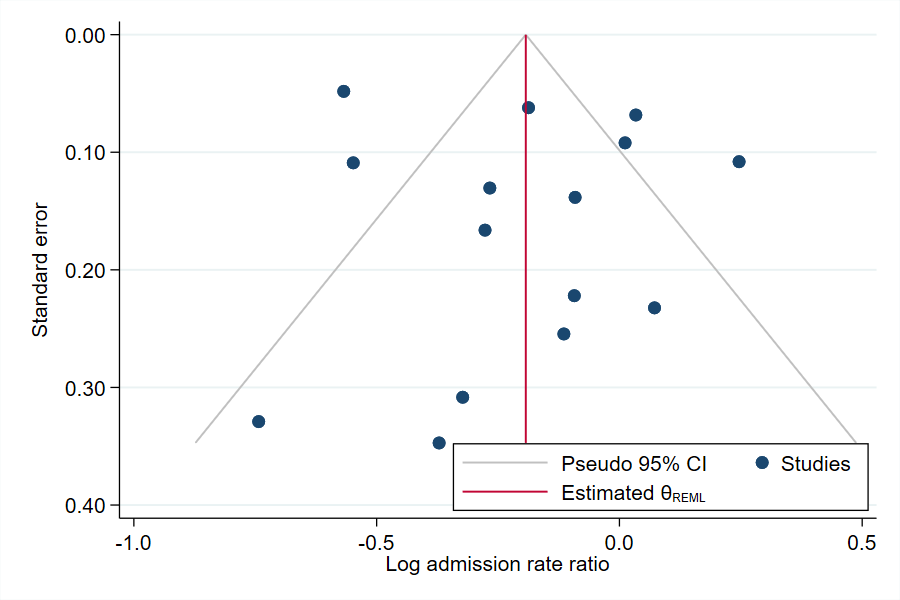


Figure 8. Funnel plot of articles studying severe stroke admissions between pandemic and pre-pandemic time-periods.


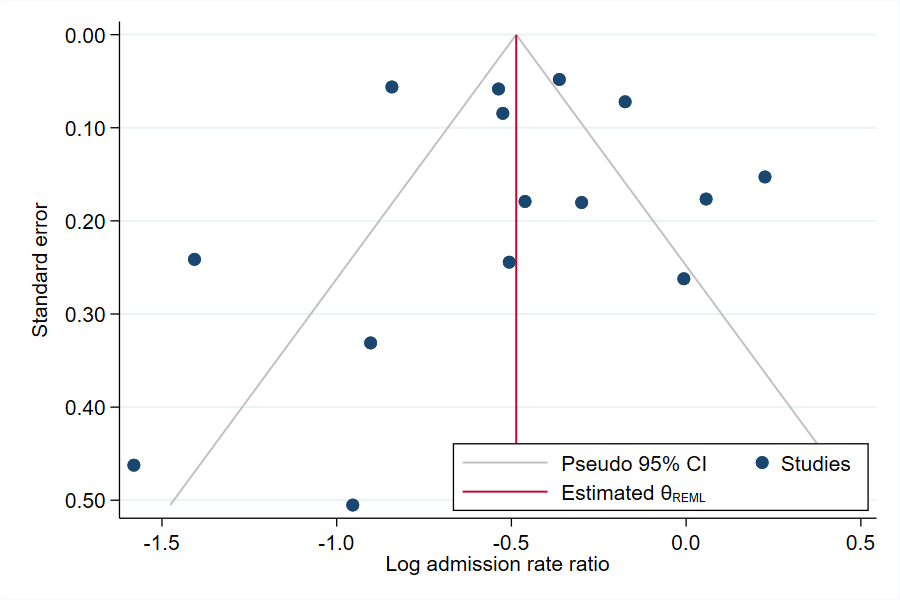


Figure 9. Funnel plot of articles studying mild stroke admissions between pandemic and pre-pandemic time-periods.


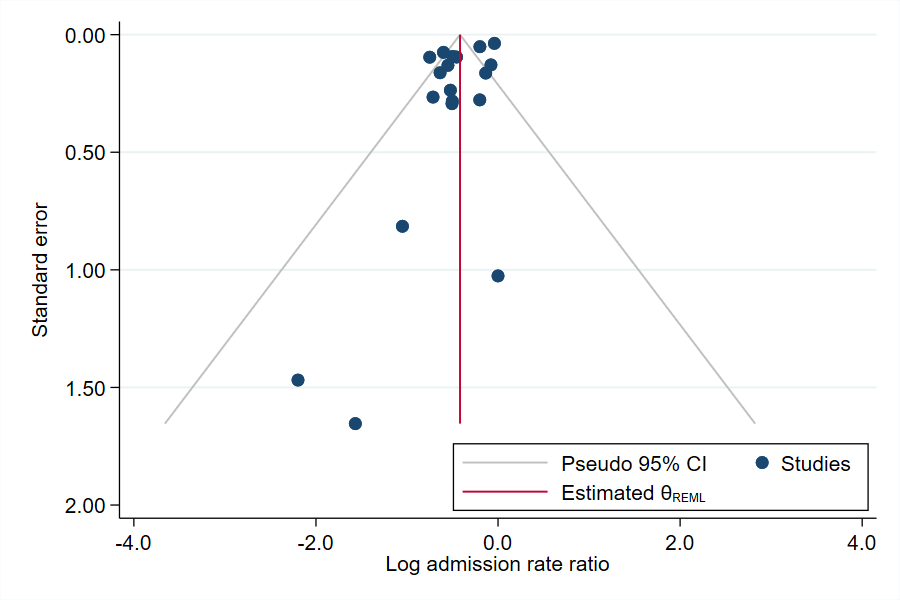


Figure 10. Funnel plot of articles studying transient ischemic attack admissions between pandemic and pre-pandemic time-periods.
