# Supplementary material for: Changes in stroke and TIA admissions during the COVID-19 pandemic: A meta-analysis
Source: Eur Stroke J. 2023 Sep 29;9(1):78–87. doi: 10.1177/23969873231204127 (PMC10916820; doi:10.1177/23969873231204127)
Supplement: sj-docx-4-eso-10.1177_23969873231204127 – Supplemental material for Changes in stroke and TIA admissions during the COVID-19 pandemic: A meta-analysis [file sj-docx-4-eso-10.1177_23969873231204127.docx]

| **PMID** | **Author** | **Control period** | **Study period** |
| --- | --- | --- | --- |
| 33758066 | B. Atchie | 1/4-30/6 2019 | 1/4-30/6 2020 |
| 33063236 | M. Balestrino | 8/3-2/5 2019 | 8/3-2/5 2020 |
| 33691503 | C. Balucani | 1/3-30/9 2019 | 1/3-30/9 2020 |
| 32755320 | J. H. Butt | 13/3-8/5 2017-2019 | 13/3-7/5 2020 |
| 33523320 | Y. G Cao | 31/12-5/5 2019 | 30/12-3/5 2020 |
| 34295099 | N. Dhar | 1/1-24/3 2020 | 25/3-30/4 |
| 32530738 | H. Diegoli | 16/2-16/3 2020 | 17/3-15/4 2020 |
| 32912527 | C. Essenwa | w1-8 2020 | w9-16 2020 |
| 33588592 | M.R. Etherton | 1/11 2019-28/2 2020 | 1/3 2020-30/4 2020 |
| 32632635 | G.Frisullo | 11/3-11/4 2019 | 11/3-11/4 2020 |
| 33738913 | A. Gabet | w12-19 2017-2019 | w12-19 2020 |
| 33185918 | Z. Gdovinova | March-April 2019 | March-April 2020 |
| 33069086 | A. Ghoreishi | 18/2-18/7 2019 | 18/2-18/7 2020 |
| 33439890 | A.T.M.H Hasan | 1/1-24/3 2020 | 25/3-16/6 2020 |
| 33011516 | S. John | 1/3-10/5 2019 | 1/3-10/5 2020 |
| 33290619 | C. Katsouras | 2/3-12/4 2019 | 2/3-12/4 2020 |
| 33536996 | J. Koge | Dec 2019-March 2020 | April 2020-July 2020 |
| 33428057 | E.S. Kristoffersen | 3/1-12/3 2020 | 13/3-30/4 2020 |
| 32510283 | M.Mehrpour | 15/2-15/4 2019 | 15/2-15/4 2020 |
| 32698917 | J.N Briard | 30/3-31/5 2019 | 30/3-31/5 2020 |
| 33039766 | N. Ohara | 3/3-25/5 2019 | 1/3-23/5 2020 |
| 33166950 | F. Rameez | 24/3-23/4 2019 | 24/3-23/4 2020 |
| 33450843 | F.M Ramirez-Moreno | 15/3-10/5 2019 | 15/3-10/5 2020 |
| 32691235 | L.A. Rinkel | 21/10-8/12 2019 | 16/3-3/5 2020 |
| 32438895 | S. Rudilosso | March 2019 | March 2020 |
| 33059543 | S. Sacco | 1/3-31/3 2019 | 1/3-31/3 2020 |
| 33814368 | E. T. Samkari | 1/1-22/3 2020 | 23/3-31/7 2020 |
| 32702560 | F.S Sarfo | Jan-June 2019 | Jan-June 2020 |
| 32855352 | M. Sharma | 31/12 2018-21/4 2019 | 30/12 2019-19/4 2020 |
| 33250041 | J.E Siegler | March-July 2019 | March-July 2020 |
| 33394194 | R. Tavanaei | 1/3-1/6 2019 | 1/3-1/6 2020 |
| 32525468 | H.T Meza | 30/12 2019-8/3 2020 | 9/3-3/5 2020 |
| 32432998 | K-C. Teo | 23/1-24/3 2019 | 23/1-24/3 2020 |
| 33049464 | J. Wang | 1/12 2019-11/3 2020 | 12/3-30/6 2020 |
| 33550778 | J. Wang | 21/1-11/3 2019 | 21/1-11/3 2020 |
| 33250851 | Y. Wu | 24/1-29/4 2019 | 24/1-29/4 2020 |
| 33672096 | K. Melaika | 1/12 2019-15/3 2020 | 16/3 2020-16/6 2020 |
| 33896223 | A. Douiri | 23/3-30/4 2017, 2018, 2019 | 23/3-30/4 2020 |
| 33789627 | J. Xin | Jan-Feb 2019 | Jan-Feb 2020 |
| 33862541 | A.N. Wallace | 1/1-29/2 2020 | 20/3-25/4 2020 |
| Embase | M. E. Tsalta-Mladenov | July-Sept 2019 | July-Sept 2020 |
| Embase | E. Altunisik | 1/4-31/5 2019 | 1/4-31/5 2020 |
| 34285718 | C. Katsouras | 1/11-31/12 2019 | 1/11-31/12 2020 |
| 34069433 | H. Chuan Loh | 1/3-30/9 2019 | 1/3-30/9 2020 |
| 34148220 | V. Raymaekers | March-May 2019 | March-May 2020 |
| 34370900 | D. Richter | March-May 2019 | March-May 2020 |
| Embase | Y. T. Tsai | 2019 | 2020 |
| 34237727 | C. Libruder | 1/1-7/3 2020 | 8/3-30/4 2020 |
| 34284323 | V. A. Pujol-Lereis | March-June 2019 | March-June 2020 |
| 34375915 | R. Bhatia | Feb-July 2019 | Feb-July 2020 |
| 34336181 | T. G. White | March-April 2019 | March-April 2020 |
| 34566833 | Q. Tan | 24/1-10/3 2019 | 24/1-10/3 2020 |
| 34247153 | S. Gu | 1/12 2019-31/1 2020 | 1/2-31/3 2020 |
| 34168026 | E. McNamara | 1/3-30/4 2019 | 1/3-30/4 2020 |
| 34515067 | P. Sedova | March-May 2019 resp March 2019 (NIHSS-data) | March-May 2020 resp April 2020 (NIHSS-data) |
| 34260632 | H. Aref | 7/12 2019-14/2 2020 | 15/2-10/5 2020 |
| 34894018 | G.M. De Marchis | 13/3-26/4 2018, 2019 | 13/3-26/4 2020 |
| 35135062 | D.A. Cadilhac | 1/1 2019 - 24/2 2020 | 25/2-23/6 2020 |
| 35324905 | N. Akhtar | Sept 2019-Feb 2020 | March-June 2020 |
